# Supplementary material for: Photocatalytic Removal of the Greenhouse Gas Nitrous Oxide by Liposomal Microreactors
Source: Angew Chem Int Ed Engl. 2022 Sep 5;61(41):e202210572. doi: 10.1002/anie.202210572 (PMC9825952; doi:10.1002/anie.202210572)
Supplement: Supplementary file 1 — Supporting Information [file ANIE-61-0-s001.pdf]

Angewandte Chemie
Eine Zeitschrift der Gesellschaft Deutscher Chemiker

Supporting Information

Photocatalytic Removal of the Greenhouse Gas Nitrous Oxide by Liposomal Microreactors

*S. E. H. Piper, C. Casadevall, E. Reisner, T. A. Clarke, L. J. C. Jeuken, A. J. Gates, J. N. Butt**

Supporting Information

EXPERIMENTAL SECTION:

Reagents: Unless otherwise stated, all chemicals were obtained from commercial suppliers and used without further purification. Ultrapure water with resistance ≥18.2 MΩ cm (Milli-Q) was used throughout. Lauryldimethylamine oxide (LDAO) was from Sigma-Aldrich, n-octyl glucoside from Anatrace, *E. coli* polar lipid extract from Avanti Polar Lipids and Triton X-100 from Acros Organics. Ethylenediaminetetraacetic acid (EDTA, 0.5 M solution) from Fisher was adjusted to pH 8.5 with 10 M NaOH prior to use. Graphitic N-doped carbon dots were prepared as previously described.[1] Briefly, these nanoparticles were synthesized by pyrolysis of aspartic acid in air at 320 °C for 100 hr. Dissolution in water and NaOH solution produced a dark brown solution, which was passed through a microfilter (0.22 µm) and freeze-dried to obtain a brown solid. Attenuated total reflectance Fourier transform infrared spectroscopy (ATR FT-IR) together with TEM characterization of the materials (Figure S5) is consistent with previously reported data.[1a]

Purification of MtrCAB was from *Shewanella oneidensis* MR-1 as described previously.[2] Prior to incorporation into liposomes, purified protein (in 2% Triton X-100, 20 mM Hepes, 150 mM NaCl, pH 7.8) was exchanged into 5 mM LDAO, 50 mM sodium phosphate, 50 mM NaCl, pH 7.5. This was achieved by detergent exchange of MtrCAB bound to a 5 mL HiTrap Q FF column (Cytiva). The column was equilibrated with 5 mM LDAO, 50 mM sodium phosphate, 50 mM NaCl, pH 7.5. Bound protein was washed (2.5 mL min^{-1}) with 5 mM LDAO, 50 mM sodium phosphate, 50 mM NaCl, pH 7.5 until the 280 nm absorbance of the eluent had fallen to a low value indicating removal of all Triton X-100 from the column (typically 15 – 20 column volumes). MtrCAB was then eluted with 0.5 M NaCl in 5 mM LDAO, 50 mM sodium phosphate, pH 7.5. Finally, a 100 kDa MWCO centrifugal filter (Millipore) was used to obtain MtrCAB in 5 mM LDAO, 50 mM sodium phosphate, 50 mM NaCl, pH 7.5. MtrCAB concentrations were calculated with the Beer-Lambert law (ε_{410nm} = 2 660 000 M^{-1} cm^{-1} for the fully oxidized (air equilibrated) protein).

Purification of N_2O Reductase (NosZ) was from *Paracoccus denitrificans* strain PD1222 containing the pMSL002 plasmid, a strain that expresses both genome encoded native NosZ and recombinant plasmid-borne NosZ with a C-terminal Strep-tag II.[3] Overexpression and purification of N_2O Reductase from anaerobic denitrifying cell cultures was performed essentially as previously described.[3] Clarified cell lysate containing biotin blocking solution (IBA Lifesciences, 10 µL per 100 mL) was loaded onto a Strep-tactin XT Superflow column (IBA Lifesciences) pre-equilibrated with 100 mM Tris:HCl, 150 mM NaCl, pH 8 (2.5 mL min^{-1}). Bound protein was washed with 10 column volumes of 100 mM Tris:HCl, 150 mM NaCl, pH 8 and eluted with 20 mM Biotin, 100 mM Tris:HCl, 150 mM NaCl, pH 8. Eluted protein was exchanged into 100 mM Tris:HCl, 150 mM NaCl, pH 8 using a 30 kDa MWCO centrifugal concentrator.

N_2O Reductase is a functional dimer containing 6 Cu atoms per monomer that are distributed within two distinct redox sites (Cu_Z and Cu_A).[4] Denaturing SDS-PAGE analysis (Figure S7A) of purified N_2O

Reductase revealed a major band at approx. 70 kDa as expected for the monomer. Liquid Chromatography Mass Spectrometry (LC-MS) (Figure S7B) performed as previously described[5] revealed a major species of mass 68,106 Da corresponding to Strep-tagged monomer (predicted mass 68 104 Da). A slightly smaller peak at 66,324 Da was attributed to the native genomically encoded monomer (predicted mass 66,322 Da). Thus, the N_2O Reductase used in these studies is most likely comprised mainly of active heterodimers containing native monomers paired with recombinant Strep-tagged monomers.

Concentrations of purified N_2O Reductase were defined using the Bradford assay calibrated with bovine serum albumin. Copper content, defined by a colorimetric bathocuproinedisulfonic acid assay,[3] was consistent with 6 ions per monomer and so consistent with full loading. Spectroscopic measurement of the oxidative decolorization of the blue methyl viologen cation radical ($MV^{•+}$) coupled to N_2O reduction, as described below, defined the turnover frequency (k_{cat}) of the activated enzyme as 20 mol N_2O per second per mol monomer.

Preparation and Quantification of N_2O Saturated Solutions: Saturated solutions of N_2O were prepared in sealed anaerobic 20 mL vials containing 5 mL of 50 mM Tris:HCl, 10 mM KCl, pH 8.5 by sparing for approximately 5 min with N_2O (99%, CK Gas Products). N_2O concentration in the aqueous phase, 15 mM, was calculated from the head space concentration by Henry's Law with the assumption of ideal gas behaviour.[1b] Head space N_2O was quantified with a Clarus 500 Gas Chromatograph fitted with an Elite-Q capillary column (30 m x 0.53 mm) and an electron capture detector. Calibration was with 100, 1 000, 5 000 and 10 000 ppm N_2O in N_2 (mol/mol) (Air Liquide). The carrier gas was oxygen-free N_2 and the make-up gas was 5% methane in argon.

Spectrophotometric Assay of N_2O Reduction by Purified N_2O Reductase. N_2O Reductase activity was routinely measured by the bleaching of blue $MV^{•+}$ (ε_{600nm} = 13 700 M^{-1} cm^{-1}) using a protocol adapted from reference [6]. Assays were performed in a N_2-filled chamber (Belle Technology, atmospheric O_2 < 2 ppm) and monitored by UV-visible spectroscopy (Jenway 7315 spectrophotometer). Purified N_2O Reductase was assayed in 1 cm path length cuvettes containing 1 mL of anaerobic 0.5 mM MV^{2+}, 50 mM Tris:HCl, 10 mM KCl, pH 8.5. An aliquot of anaerobic dithionite solution was added to give a stable absorbance at 600 nm of approximately 1.0 due to the presence of blue $MV^{•+}$. An aliquot of enzyme was then added to the cuvette (to give approximately 25 nM) and allowed to activate[6] for 5 min. Finally, an aliquot of saturated N_2O solution was added (final concentration approximately 750 µM N_2O in the aqueous phase). N_2O reduction coupled to oxidative decoloration of the $MV^{•+}$ was quantified by the absorbance decrease at 600 nm.

Proteoliposome Preparation. Proteoliposomes with encapsulated N_2O Reductase and MtrCAB in the lipid bilayer were prepared in parallel to proteoliposomes with encapsulated enzyme and no MtrCAB. For this two samples of polar lipid extract (20 mg) were dispersed in separate 500 µL volumes of 50 mM Tris:HCl, 10 mM KCl, pH 8.5 by vortex for 30 min at room temperature. Then each of the following steps was followed by gentle mixing unless stated otherwise. To each sample was added 200 µL of 0.5 M octyl glucoside, 50 mM Tris:HCl, 10 mM KCl, pH 8.5. Samples were then left on ice for 10 min to solubilize the lipid. To prepare liposomes containing MtrCAB, a sample of soluble lipid was mixed with 100 µL of 25 µM MtrCAB in 50 mM sodium phosphate, 50 mM NaCl, 5 mM LDAO, pH 7.5. For liposomes without MtrCAB, soluble lipid was mixed with 100 µL of 50 mM sodium phosphate, 50 mM NaCl, 5 mM LDAO, pH 7.5. Both samples were left on ice for 10 min, then 125 µL of 400 µM N_2O Reductase, 100 mM Tris:HCl, 150 mM NaCl, pH 8.5 was added. The samples were kept on ice for a further 10 min, then added to 100 mg Bio-Bead SM-2 Resin (Bio-Rad) and incubated on ice for 30 min with occasional inversion. After the resin had settled under gravity, the solution was recovered and transferred to fresh Bio-Bead SM-2 Resin (100 mg) and this process was repeated three times for a total of four 30 min incubation steps. The formation of proteoliposomes was triggered by adsorption of octyl glucoside onto the Bio-Bead SM-2 Resin and revealed by an increased cloudiness of the solutions.

To recover proteoliposomes and remove N_2O Reductase from the surrounding solution, the samples were first diluted to 24 mL with 100 mM Tris:HCl, 150 mM NaCl, pH 8.5. Proteoliposomes were then pelleted by ultracentrifugation (430 000 x *g*, 30 min, 4°C). After transfer to a N_2-filled chamber the supernatant was discarded, the proteoliposomes resuspended in 25 mL of anaerobic 100 mM Tris:HCl, 150 mM NaCl, pH 8.5, and the resulting solution sealed inside clean ultracentrifuge tubes. These steps were repeated (typically 3 ultracentrifugation steps in total) until the supernatant was free of protein as judged by Bradford assay. At this stage the proteoliposomes were resuspended in 1 mL of anaerobic 100 mM Tris:HCl, 150 mM NaCl, pH 8.5 and any aggregated materials removed from the suspension prior to use by centrifugation at 3000 x *g* (3 min, ambient temperature). The proteoliposome

concentration in the resulting samples was approximately 300 nM as estimated from their dimensions and lipid composition.[1b]

Proteoliposome dimensions and zeta potentials were measured with a Zetasizer Nano with DTS1070-folded capillary cells (Malvern Panalytical). Samples contained approximately 15 nM proteoliposome, 50 mM Tris:HCl, 10 mM KCl, pH 8.5 and were equilibrated at 25°C for 2 min prior to measurements. Solvent viscosity was taken to be that of water. The presence of proteins in each sample was assessed using SDS-PAGE with proteins visualized by peroxidase-linked heme stain[7] or Coomassie stain.

MtrCAB content was estimated by UV-visible electronic absorption spectroscopy of samples containing approximately 5 nM proteoliposome using an extinction coefficient at 410 nm of 2 660 000 M^{-1} cm^{-1} for the fully oxidized (air equilibrated) protein. Prior to use of the Beer-Lambert law the contribution due to liposome scattering was estimated (as = A + B/λ^3 with the variables A and B adjusted to give good fit to the data) and subtracted from the measured data. N_2O Reductase could not be measured by absorption spectroscopy due to its low extinction coefficients across visible wavelengths.[8] Instead N_2O Reductase activity was measured after lysing open the proteoliposomes. For these measurements anaerobic cuvettes contained 0.5% (v/v) Triton X-100 to lyse the liposomes, 0.5 mM methyl viologen (MV^{2+}), 50 mM Tris:HCl, 10 mM KCl, pH 8.5. Sufficient sodium dithionite was added to produce an absorbance at 600 nm of ≈1.1. Proteoliposomes were then added (10 μL of approx. 300 nM to give a concentration in the cuvette of 3 nM) and after approximately 1 min N_2O was introduced (50 μL of N_2O saturated solution to give 750 μM N_2O in the cuvette). Rates of N_2O Reductase activity were quantified through bleaching of the dithionite reduced $MV^{\bullet+}$ as described above for the purified enzyme.

Measurement of N_2O Reductase Activity in Intact Proteoliposomes. Spectroscopic quantification of the N_2O Reductase activity of intact proteoliposomes was by the oxidation of sodium dithionite using an extinction coefficient[9] at 315 nm of 8 000 M^{-1} cm^{-1}. Assays were performed in a N_2-filled chamber (Belle Technology, atmospheric O_2 < 2 ppm) and monitored by electronic absorbance spectroscopy (Jenway 7315 spectrophotometer). An aliquot of liposomes (10 µL of 300 nM) was introduced to a sealed anaerobic 1 cm path length cuvette containing 1 mL of sodium dithionite (approx. 0.1 mM to give an absorbance at 315 nm of approx. 0.8), 0.01 mM MV^{2+}, 50 mM Tris:HCl, 10 mM KCl, pH 8.5. After 1 min N_2O was introduced to give a solution concentration of 0.75 mM and spectra recorded for 12 min. The time course of $MV^{\bullet+}$ oxidation was defined using ε_{395nm} = 40 000 M^{-1} cm^{-1}.

Light-driven assays of N_2O Reductase activity in intact proteoliposomes used graphitic N-doped carbon dots as photosensitiser and ethylene diamine tetraacetic acid (EDTA) as sacrificial electron donor. Graphitic N-doped carbon dots were prepared as previously described.[1] N_2O concentrations were measured with a Clarus 500 Gas Chromatograph fitted with an Elite-Q capillary column (30 m x 0.53 mm) and an electron capture detector as described above. For the assays, carbon dots were first suspended to 1 mg ml^{-1} in anaerobic 50 mM Tris:HCl, 10 mM KCl, pH 8.5. Assays were performed anaerobically in 3 mL exetainer vials containing 2 mL of a suspension of 100 µg mL^{-1} carbon dots in 0.01 mM MV^{2+}, 25 mM EDTA, 50 mM Tris:HCl, 10 mM KCl, pH 8.5. Then 1.5 µmol N_2O were introduced as an aliquot of an N_2O saturated solution in 50 mM Tris:HCl, 10 mM KCl, pH 8.5. The vials were equilibrated overnight, at which time gas chromatography found the headspace N_2O concentrations were approx. 650 µM, e.g., Figure S6A. Proteoliposomes were introduced into the vials to a final concentration of approx. 8 nM and the vials were irradiated for 4 hr by visible light (λ > 400 nm) from the side using a Krüss cold light source with a fiber optic light pipe as described in Rowe *et al*.[10]). Light intensity was measured at 2.5 kW m^{-2} using an Amprobe Solar-100 solar power meter. Samples were gently inverted at 30 min intervals. Headspace samples (100 µL) were transferred to separate 3 mL N_2-filled exetainer storage vials immediately before, and 1, 2, 3, 4 and 8 hr after addition of liposomes. Samples (50 µL) from these storage vials were analysed by gas chromatography which allowed N_2O headspace concentrations to be determined for the proteoliposome containing vials.

Separate experiments were performed to understand the time taken for headspace N_2O concentrations to respond to a change of N_2O concentration in solution. For these experiments 1.5 µmol N_2O, as an aliquot of N_2O saturated solution, was introduced into anaerobic 3 mL exetainer vials containing 1 mL headspace and 2 mL of 50 mM Tris:HCl, 10 mM KCl, pH 8.5. The vials were equilibrated overnight, at which time gas chromatography found the headspace N_2O concentrations were approx. 650 µM, e.g., Figure S6B. Aliquots of MV and sodium dithionite were added to give 1600 µM and 800 µM final concentrations respectively, the latter being slight excess with respect to total N_2O. Sufficient N_2O Reductase (150 nM) to reduce all the N_2O within 5 min was then added to half of the vials. Headspace

samples (100 µL) from all vials were transferred to separate N_2-filled exetainers after 20, 40, 60 and 120 min. Gas chromatography of the extracted sample headspaces defined their N_2O concentrations.

References:

[1] a) B. C. M. Martindale, G. A. M. Hutton, C. A. Caputo, S. Prantl, R. Godin, J. R. Durrant, E. Reisner, *Angew. Chem. Int. Ed.* **2017**, *56*, 6459-6463; b) S. E. H. Piper, M. J. Edwards, J. H. van Wonderen, C. Casadevall, A. Martel, L. J. C. Jeuken, E. Reisner, T. A. Clarke, J. N. Butt, *Front. Microbiol.* **2021**, *12*, 714508.

[2] C. W. J. Lockwood, J. H. van Wonderen, M. J. Edwards, S. E. H. Piper, G. F. White, S. Newton-Payne, D. J. Richardson, T. A. Clarke, J. N. Butt, *Methods Enzymol.* **2018**, *613*, 257-275.

[3] S. P. Bennett, M. J. Soriano-Laguna, J. Bradley, D. A. Svistunenko, D. J. Richardson, A. J. Gates, N. E. Le Brun, *Chem. Sci.* **2019**, *10*, 4985-4993.

[4] A. Pomowski, W. G. Zumft, P. M. H. Kroneck, O. Einsle, *Nature* **2011**, *477*, 234-237.

[5] L. P. Jenner, J. M. Kurth, S. van Helmont, K. P. Sokol, E. Reisner, C. Dahl, J. M. Bradley, J. N. Butt, M. R. Cheesman, *J. Biol. Chem.* **2019**, *294*, 18002-18014.

[6] S. Ghosh, S. I. Gorelsky, P. Chen, I. Cabrito, J. J. G. Moura, I. Moura, E. I. Solomon, *J. Am. Chem. Soc.* **2003**, *125*, 15708-15709.

[7] P. E. Thomas, D. Ryan, W. Levin, *Anal. Biochem.* **1976**, *75*, 168-176.

[8] T. Rasmussen, B. C. Berks, J. N. Butt, A. J. Thomson, *Biochem. J.* **2002**, *364*, 807-815.

[9] M. Dixon, *Biochim. Biophys. Acta* **1971**, *226*, 241-258.

[10] S. F. Rowe, G. Le Gall, E. V. Ainsworth, J. A. Davies, C. W. J. Lockwood, L. Shi, A. Elliston, I. N. Roberts, K. W. Waldron, D. J. Richardson, T. A. Clarke, L. J. C. Jeuken, E. Reisner, J. N. Butt, *ACS Catal.* **2017**, *7*, 7558-7566.

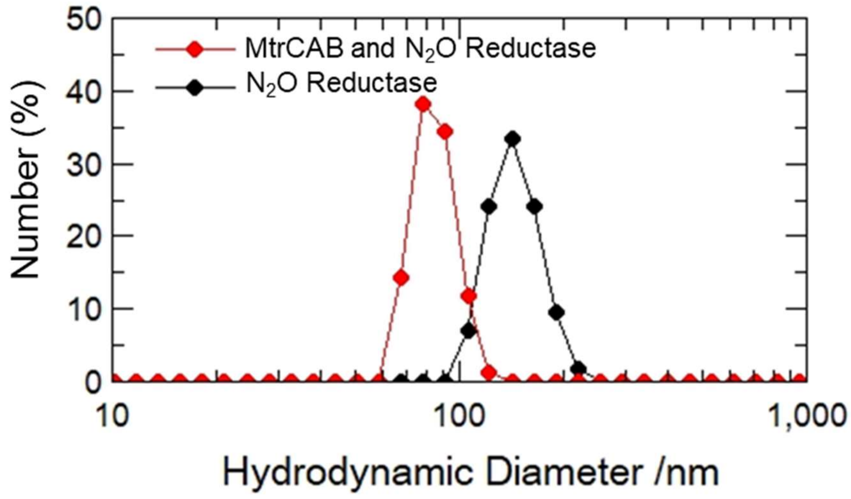

Figure S1. Size Analysis of Proteoliposomes. Dynamic light scattering of N_2O Reductase containing proteoliposomes prepared in the presence (red circles) and absence (black circles) of MtrCAB. Lines provide a guide to the eye. Samples contained approximately 15 nM proteoliposomes in 50 mM Tris:HCl, 10 mM KCl, pH 8.5.

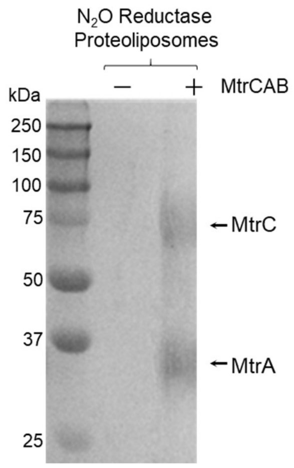

Figure S2. SDS-PAGE Analysis of Proteoliposomes. Gel images for N_2O Reductase containing proteoliposomes with and without MtrCAB as indicated. Proteins visualized by peroxidase-linked heme stain. Molecular weight markers of the indicated mass (left lane).

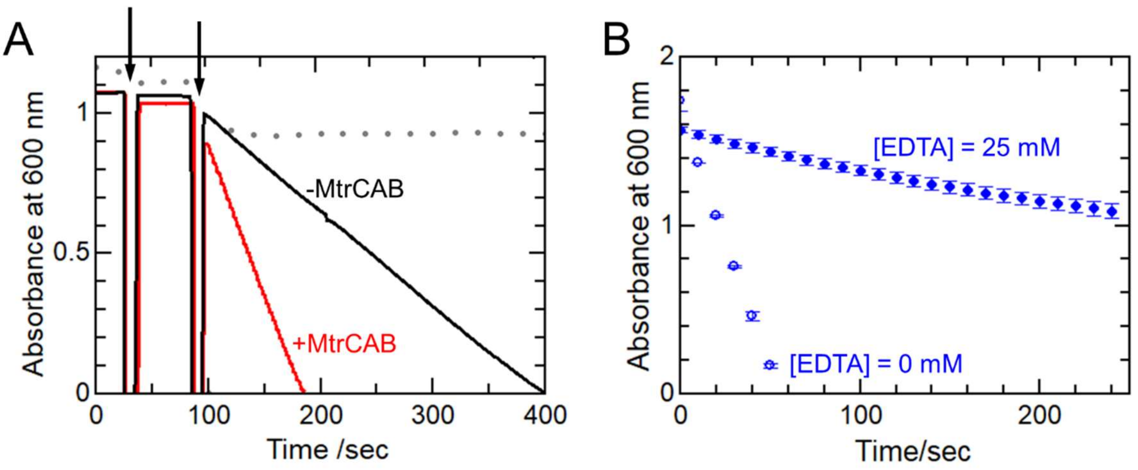

Figure S3. Spectrophotometric Assays of N_2O Reductase Activity. Oxidation of $MV^{\bullet+}$ (600 nm absorbance) coupled to N_2O reduction for:

(A) lysed N_2O Reductase containing proteoliposomes (approx. 3 nM) prepared with (red) and without (black) MtrCAB. Cuvettes contained anaerobic 0.5% (v/v) Triton X-100 to lyse the proteoliposomes, 0.5 mM MV, 50 mM Tris:HCl, 10 mM KCl, pH 8.5 with sufficient sodium dithionite to produce an absorbance at 600 nm of ≈1.1. Arrows indicate addition of proteoliposomes followed by an aliquot of N_2O saturated solution to give 750 μM N_2O. Gray circles are from an equivalent experiment with addition of anaerobic buffer in place of proteoliposomes.

(B) N_2O Reductase (7.5 nM) incubated for 10 min with approx. 120 μM $MV^{\bullet+}$ and the indicated EDTA concentration prior to addition of an aliquot of N_2O saturated solution to give 750 μM N_2O at t = 0. Cuvettes contained anaerobic 0.5 mM MV, 50 mM Tris:HCl, 10 mM KCl, pH 8.5 with sufficient sodium dithionite to produce an absorbance at 600 nm of ≈1.7. Circles show the average of *n* = 3 data sets with error bars as standard deviation.

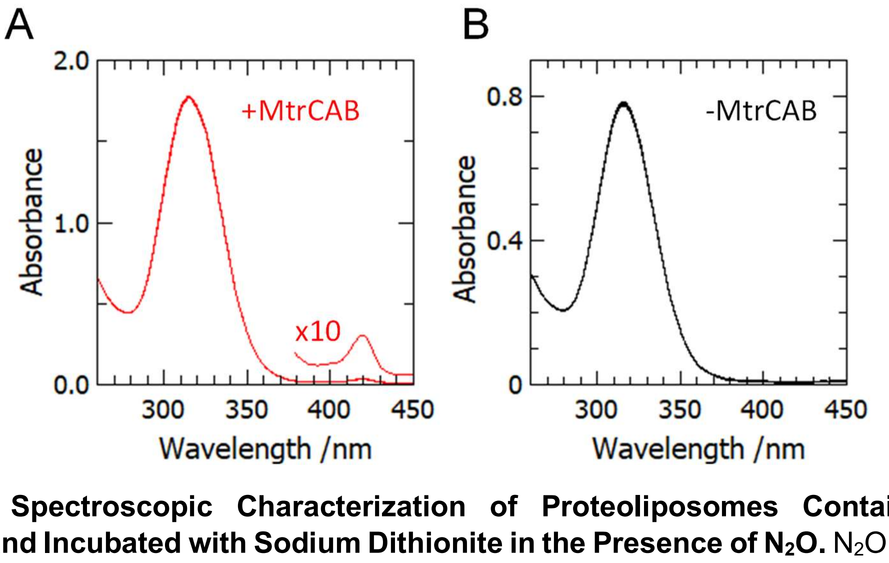

Figure S4. Spectroscopic Characterization of Proteoliposomes Containing N_2O Reductase and Incubated with Sodium Dithionite in the Presence of N_2O. N_2O Reductase containing proteoliposomes were incubated with sodium dithionite and 750 μM N_2O in anaerobic 50 mM Tris-HCl, 10 mM KCl, pH 8.5. Spectra were collected over 20 min and are displayed overlaid for (A) 220 μM sodium dithionite and 6 nM proteoliposomes containing MtrCAB and (B) 110 μM sodium dithionite and 3 nM proteoliposomes without MtrCAB. For (A) the region reporting the Soret peak from MtrCAB is also shown ×10 magnification.

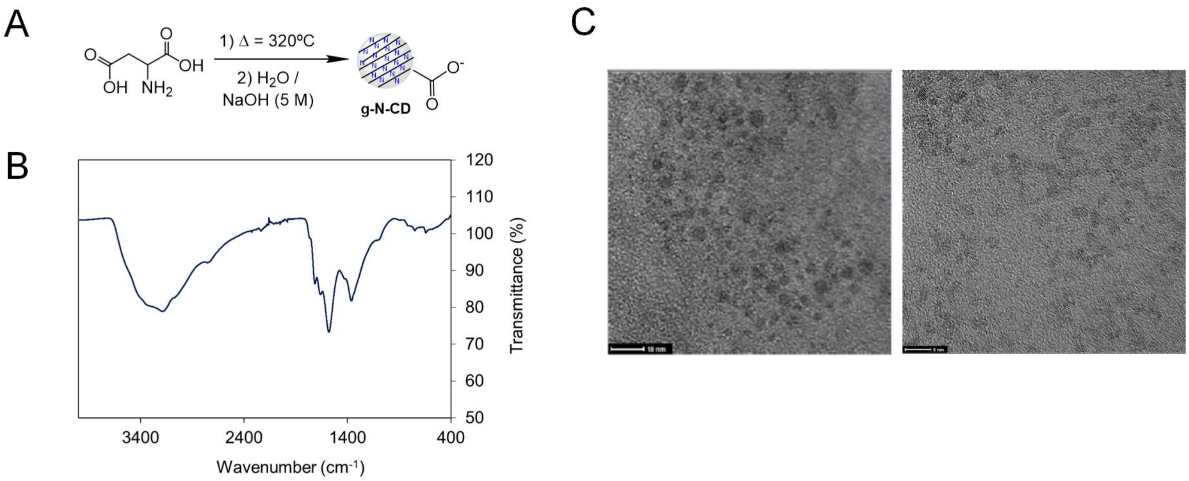

Figure S5. Description of Graphitic N-doped Carbon Dots. (A) Synthesis of graphitic N-doped carbon dots by pyrolysis of aspartic acid.[1a] (B) ATR-FTIR spectrum of graphitic N-doped carbon dots used in this study. (C) Transmission electron microscopy images of graphitic N-doped carbon dots used in this study. Scale bar 10 nm (left) and 5 nm (right). Images were collected on a Thermo Scientific (FEI) Talos F200X G2 TEM machine at an accelerating voltage of 200 kV. The images show the carbon dots have a diameter of 3.1 ± 1.1 nm consistent with those reported by Martindale *et al.*[1a] A molecular weight of 21 000 g mol^{-1} is calculated based on the density of graphite (2.266 g cm^{-3}).

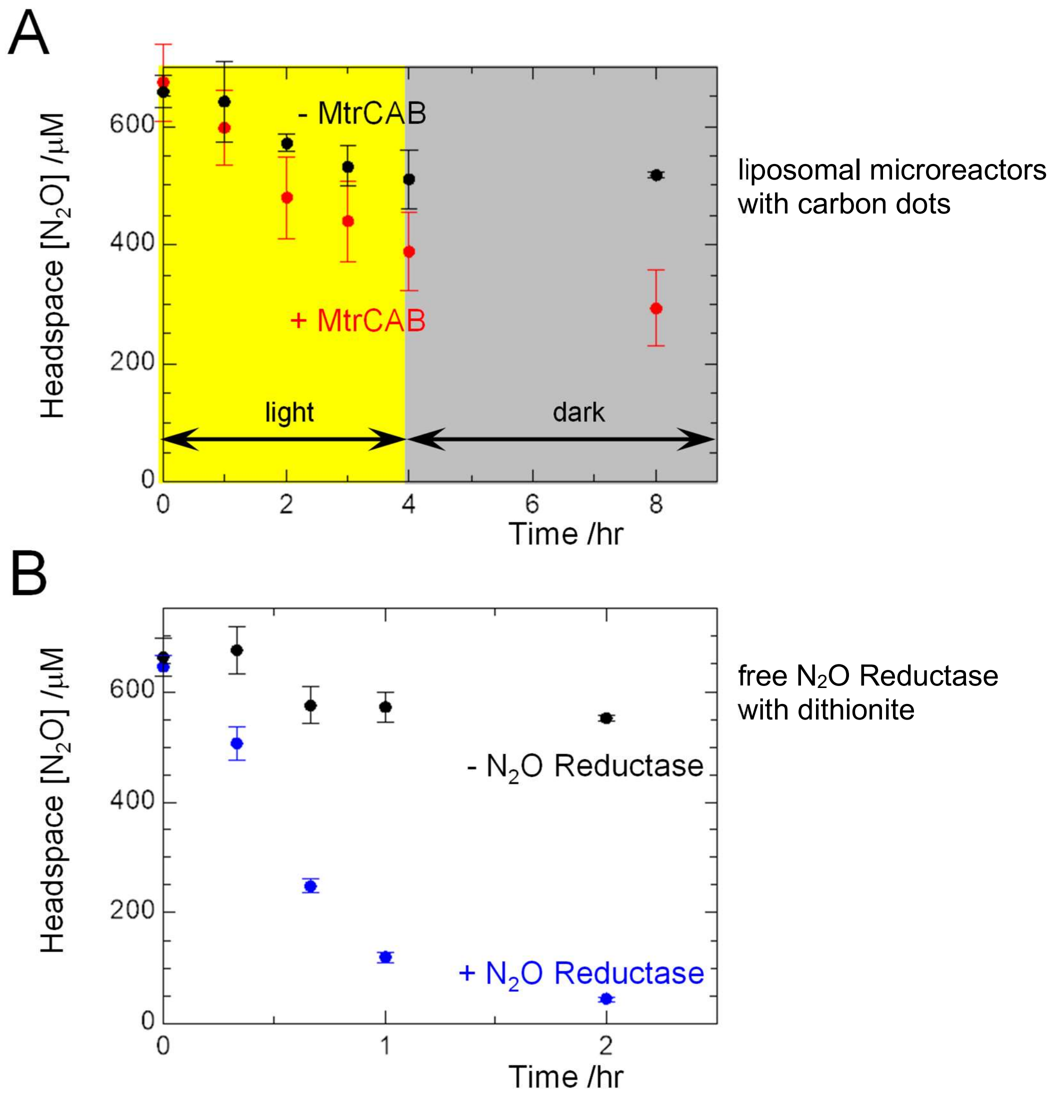

Figure S6. Headspace N_2O Quantified by Gas Chromatography.

(A) Suspensions of N_2O Reductase containing proteoliposomes (3 nM) with (red) and without (black) MtrCAB. Proteoliposomes were added at t = 0 hr to anaerobic vials containing 1.5 µmol N_2O equilibrated across 1 mL N_2 headspace and 2 mL of 100 µg mL^{-1} graphitic N-doped carbon dots, 10 µM MV, 25 mM EDTA, 50 mM Tris-HCl, 10 mM KCl, pH 8.5. Irradiation with visible light (2.5 kW m^{-2}) 0 to 4 hr was followed by 4 hr darkness. Samples were removed for analysis at the indicated times such that a small, but not insignificant, quantity of N_2O was removed from the system at these times, see Experimental Section. Thus, the N_2O concentration decreased over time even in the absence of MtrCAB. Circles show the average of n = 3 data sets with error bars as standard deviation.

(B) Samples with (blue) and without (black) N_2O Reductase (150 nM) added at t = 0 hr. Anaerobic vials contained 1.5 µmol N_2O equilibrated across 1 mL N_2 headspace and 2 mL of 1600 µM MV, 800 µM dithionite, 50 mM Tris:HCl, 10 mM KCl, pH 8.5. Complete removal of N_2O was expected in 5 min when N_2O Reductase was present. Samples were removed for analysis at the indicated times such that a small, but not insignificant, quantity of N_2O was removed from the system at these times, see Experimental Section. Thus, the N_2O concentration decreased over time even though no gas was reduced in the absence of N_2O Reductase. Circles show the average of n = 3 data sets with error bars as standard deviation.

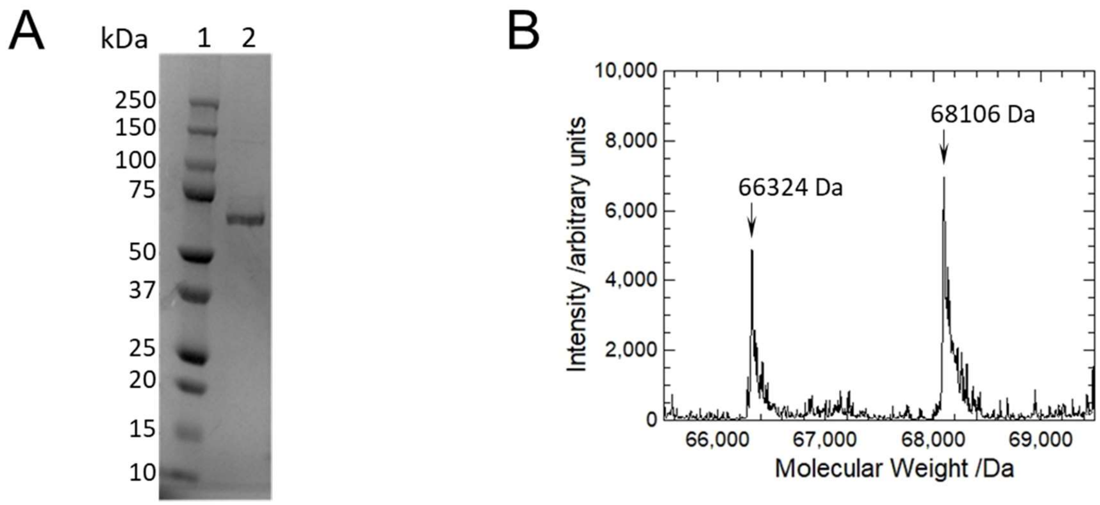

Figure S7. Characterization of N_2O Reductase. (A) SDS-PAGE image for purified N_2O Reductase with protein visualized by Coomassie stain. Molecular weight markers (lane 1) and N_2O Reductase (lane 2). (B) LC-MS analysis of purified N_2O Reductase. The feature corresponding to 66 324 Da is attributed to genomically encoded protein. The feature corresponding to 68 106 Da is attributed to plasmid encoded Strep II-tagged protein. See Experimental Section for details.
